# Determinantes sociales de la salud mental: políticas públicas desde el modelo biopsicosocial en países latinoamericanos

**DOI:** 10.26633/RPSP.2021.158

**Published:** 2021-12-16

**Authors:** Varinia Leiva- Peña, Patricia Rubí-González, Benjamin Vicente-Parada

**Affiliations:** 1 Universidad de Concepción, Facultad de Medicina, Departamento de Psiquiatría y Salud Mental Concepción Chile; 2 Universidad de Concepción, Facultad de Medicina, Departamento de Psiquiatría y Salud Mental Concepción Chile; 3 Universidad de Concepción, Facultad de Medicina, Departamento de Psiquiatría y Salud Mental Concepción Chile

**Keywords:** Salud mental, modelos biopsicosociales, política de salud, determinantes sociales de la salud, Mental health, models, biopsychosocial, health policy, social determinants of health, Saúde mental, modelos biopsicossociais, política de saúde, determinantes sociais da saúde

## Abstract

En este artículo se expone la evidencia sobre la implementación de políticas públicas en salud mental, con el objetivo de describir los avances y los desafíos para poner en marcha el modelo biopsicosocial y comunitario principalmente en Latinoamérica. Se realizó una revisión teórica de artículos indexados en Web Of Science, Scopus, PubMed y SciELO. Se incorporaron informes y programas gubernamentales. Los determinantes sociales impactan a la salud mental, la pobreza aumenta significativamente el riesgo de desarrollar una psicopatología. Por ello, la Organización Mundial de la Salud insiste en la necesidad de adoptar un paradigma biopsicosocial para enfrentar los desafíos de salud mental. Alrededor de un tercio de los países, todavía no cuenta con una política nacional de salud mental y existen grandes disparidades de financiamiento y cobertura para la población entre países de ingresos altos y bajos. Particularmente de Latinoamérica, los resultados ilustran un avance en países de ingresos medios y bajos, en elaborar programas de salud mental desde un enfoque comunitario y biopsicosocial. Sin embargo, presentan desafíos en su operacionalización, financiamiento y adaptación a sus realidades socioculturales. La evidencia indica que para avanzar desde un paradigma biomédico hacia uno que incorpore los determinantes sociales de la salud, no se deberían mantener idénticas estrategias en comunidades distintas, puesto que las necesidades de países de ingresos bajos y medios difieren a las de países de ingresos altos. Por ello, resulta fundamental aumentar la investigación local para generar evidencia que refleje las necesidades nacionales en materia de políticas públicas en salud mental.

## Abstract (en)

This article presents evidence on the implementation of public policies on mental health, and describes the advances and challenges to implementing the biopsychosocial and community model, mainly in Latin America. A theoretical review was conducted of articles indexed in Web of Science, Scopus, PubMed and SciELO. Government reports and programs were included. Social determinants impact mental health. In particular, poverty significantly increases the risk of developing a mental disorder. Accordingly, the World Health Organization insists on the need to adopt a biopsychosocial paradigm to address mental health challenges. About a third of countries still do not have a national mental health policy and there are large disparities in funding and population coverage between high- and low-income countries. Particularly in Latin America, the results illustrate progress in low- and middle-income countries in terms of developing mental health programs with a community and biopsychosocial approach. However, there are challenges in their operationalization, financing, and adaptation to socio-cultural realities. The evidence indicates that in order to move from a biomedical paradigm to one that incorporates the social determinants of health, identical strategies should not be employed in all communities, since the needs of low- and middle-income countries differ from those of high-income countries. Therefore, it is essential to increase local research to generate evidence that reflects national needs with regard to public policies on mental health.

## RESUMO

Este artigo apresenta evidências sobre a implementação de políticas públicas de saúde mental, com o objetivo de descrever os avanços e desafios para a implementação do modelo biopsicossocial e comunitário, principalmente na América Latina. Foi realizada uma revisão teórica de artigos indexados nas plataformas Web of Science, Scopus, PubMed e SciELO e foram incluídos relatórios e programas governamentais. Os determinantes sociais afetam a saúde mental, e a pobreza aumenta significativamente o risco de desenvolvimento de psicopatologias. Por essa razão, a Organização Mundial da Saúde insiste na necessidade de se adotar um paradigma biopsicossocial para enfrentar os desafios da saúde mental. Aproximadamente um terço dos países ainda não conta com uma política nacional de saúde mental e existem grandes disparidades de financiamento e cobertura para a população entre países de alta e baixa renda. Particularmente na América Latina, os resultados mostram um avanço em países de baixa e média renda na elaboração de programas de saúde mental a partir de um enfoque comunitário e biopsicossocial. No entanto, apresentam desafios na operacionalização, financiamento e adaptação a suas realidades socioculturais. As evidências indicam que, para passar de um paradigma biomédico a outro que inclua os determinantes sociais da saúde, não deveriam ser mantidas as mesmas estratégias em comunidades distintas, uma vez que as necessidades dos países de baixa e média renda diferem das dos de alta renda. Portanto, é fundamental ampliar a pesquisa local para gerar evidências que reflitam as necessidades nacionais de políticas públicas de saúde mental.

Los trastornos de salud mental son altamente prevalentes en el mundo, principalmente en países de ingresos medios y bajos (1). La Organización Mundial de la Salud (OMS) define la salud como un estado de bienestar físico, social y mental, el cual no es únicamente determinado por la ausencia de patología, sino también por la promoción de una salud integral y de bienestar (2). De esta manera, la salud, debiera ser abordada en función de su complejidad y no solo desde un enfoque biomédico.

La salud se ve influenciada por las condiciones biopsicosociales en las que se desenvuelven las personas. Por ello, en el 2005, la OMS estableció la Comisión sobre Determinantes Sociales de la Salud, los cuales son definidos como aquellas condiciones en que las personas viven, nacen, crecen, envejecen, trabajan y se desenvuelven cotidianamente, incluido su sistema de salud. Estas circunstancias impactan en la salud y explican muchas de las inequidades sanitarias existentes, que aumentan o disminuyen el riesgo de enfermar (3).

Consecuentemente, la salud mental también se ve influida por los determinantes sociales, de los cuales la pobreza e inequidad socioeconómica están fuertemente relacionadas a un mayor riesgo de desarrollar psicopatología (4). Así, en virtud de los factores socioambientales asociados a la etiología de enfermedad mental, existen dos hipótesis predominantes de mecanismos que vinculan al trastorno mental con la desventaja socioeconómica, estos son las hipótesis de selección social y de causalidad social (5). La primera indica que personas con algún tipo de predisposición genética a patología mental o somática, luego de enfermar, descienden de la escala social en la que estaban previamente. La segunda, argumenta que la situación social por sí misma causaría enfermedad mental. Existe evidencia que sustenta la hipótesis de la causalidad social, asociando pobreza o vulnerabilidad social a mayor probabilidad de desarrollar trastornos mentales (6). Si bien hay psicopatología desarrollada debido a su alta carga genética, como por ejemplo la esquizofrenia, en otras enfermedades como la depresión, el trastorno obsesivo compulsivo o el abuso de sustancias, la enfermedad se debería fundamentalmente a dinámicas socioambientales (6).

Diversos expertos destacan la importancia de adoptar un paradigma biopsicosocial para enfrentar los desafíos de salud mental de la población. Saraceno (6) plantea que, en la cultura occidental se han consolidado estilos de vida que incrementan la probabilidad de enfermar psíquica y físicamente. La pobreza, la deprivación, los hábitos de consumo, el estrés y los modos de vida poco saludables contribuyen al aumento de enfermedades de causalidad social y de carácter crónico, que son complejas y requieren ser abordadas holísticamente, en lugar de manejarlas primordialmente desde el modelo biomédico.

Las patologías crónicas, requieren de intervención constante y prolongada en el tiempo, quedando el modelo farmacológico tradicional insuficiente para responder a la compleja etiología y curso de estas enfermedades. Dentro de las enfermedades crónicas, los trastornos mentales y las enfermedades no transmisibles serían las de mayor frecuencia y aumento durante las últimas décadas (6). Para enfrentarlas, Saraceno (6) propone modelos integrales, tanto para su tratamiento, como para su prevención y promoción, indicando que la salud pública debiese tomar un rol protagónico y superar el modelo biomédico tradicional, fortaleciendo la intervención comunitaria, así como el empoderamiento de los pacientes, para hacerlos activos en sus procesos sanitarios. Sugiere fortalecer la atención primaria y secundaria, como también sus lazos con la comunidad y servicios comunitarios, a fin de superar el modelo hospitalario de atención terciaria y del hospital psiquiátrico. Argumenta que el hospital general, cumpliría una función vicaria y sedante para el manejo de la enfermedad y por su parte, el manicomio, históricamente ha desarrollado un rol residencial y marginalizador para las personas con trastornos mentales; ambos operan desde una perspectiva reduccionista, sin exhibir resultados efectivos en el tratamiento de la enfermedad psíquica (6).

Saraceno (6) refiere que, a nivel mundial todavía estaríamos lejanos a superar el modelo biomédico y hospitalocéntrico, pero que la investigación y algunas experiencias de carácter local y en países de renta alta aportan evidencia preliminar de la efectividad de este enfoque y la necesidad de abordar esta problemática de salud pública de manera integral. Propone que el personal de salud primaria debiese estar mínimamente entrenado en temáticas prácticas y teóricas de salud mental, así como, involucrar a la comunidad a través de actividades promocionales y de hábitos que potencien la salud mental comunitaria y educación en este ámbito, favoreciendo tanto su empoderamiento como su conocimiento. Plantea igualmente una integración entre el sistema primario y secundario de salud, para intervenir coordinadamente en casos más complejos, pero desde esta perspectiva integral y socio-comunitaria. Para ello sugiere, por ejemplo, los denominados “Task Shifting”, es decir, la delegación a personal menos calificado de ciertas atribuciones e intervenciones, originalmente pensadas para personal más profesionalizado. Esta práctica se desarrolla en donde los recursos humanos cualificados son escasos, por lo que, mediante capacitaciones técnicas y prácticas en salud mental, se potencia el capital humano existente, implementando estrategias de intervención psicosocial que favorecen el manejo de casos de mediana complejidad en lugar de ser derivados inmediatamente al sistema terciario, posiblemente colapsado (6).

Este artículo expone la evidencia sobre la implementación de programas y políticas públicas en salud mental de carácter internacional, con el objetivo de describir los avances y desafíos, para la implementación del modelo biopsicosocial y comunitario principalmente en Latinoamérica.

## MÉTODOS

Se realizó una revisión teórica de artículos indexados en las bases de datos Web of Science, Scopus, PubMed y SciELO. Las palabras claves fueron “Salud Mental”, “Políticas Públicas”, “Salud Pública” y “Biopsicosocial”, priorizándose publicaciones entre 2017 y 2020. Igualmente, se incorporaron informes y programas gubernamentales de salud mental, artículos y literatura asociada a políticas públicas y datos epidemiológicos en salud mental. Se priorizaron documentos que dieran cuenta de políticas públicas en Latinoamérica.

## RESULTADOS Y DISCUSIÓN

Respecto a las políticas públicas y salud mental, la evidencia tiende a concluir que los desafíos en mejorar los programas y políticas de salud mental guardan relación con la integración y comprensión de sus determinantes sociales y multicausalidad en la etiología de la enfermedad mental, así como de la adecuación de los mismos a las distintas realidades y necesidades locales (7). Alrededor de un tercio de los países todavía no cuentan con una política pública nacional de salud mental y existen grandes disparidades en las tasas de cobertura para la población entre países de ingresos altos y bajos, así como diferencias presupuestarias para políticas públicas en salud mental (3), como lo ilustran los cuadros 1 y 2. En el Plan de Acción para Salud Mental 2013-2020 de la OMS (8), se sugiere la desinstitucionalización progresiva de los programas nacionales de salud mental, para focalizar las estrategias en intervenciones comunitarias. A pesar a estas sugerencias, a nivel internacional todavía el 67% de los recursos de salud mental se mantienen focalizados en los hospitales psiquiátricos (6).

Pese a que hay países que en sus planes nacionales de salud mental han incorporado progresivamente un enfoque biopsicosocial, países de altos ingresos exhiben necesidades distintas a países de ingresos bajos y medios. Un estudio concluye que países de rentas altas tienden a presentar problemáticas asociadas a las dificultades pragmáticas de virar desde el ejercicio biomédico hacia el biopsicosocial (9). Por su parte, países de ingresos bajos y medios, enfrentan dificultades principalmente relacionadas al financiamiento y escasez de políticas públicas en salud mental, dependiendo asimismo de financiamiento internacional (9). Igualmente, estos países suelen replicar programas aplicados en países de altos ingresos, los cuales no responden necesariamente a sus necesidades reales (10). Existe evidencia que desde la década del 2000 hay un incremento de estudios en salud mental en países en vías de desarrollo, pero éstos parecieran referirse principalmente a la implementación de programas de experiencias anteriores en países de altos ingresos (9). Respecto a las enfermedades mentales más frecuentemente estudiadas, destacan en primer lugar la depresión, seguida de estudios de estrés y psicosis (11).

**CUADRO 1.** Presupuesto en Salud y antecedentes sociodemográficos según países seleccionados.

País	Porcentaje de gasto en salud en relación al PIB según el Banco Mundial en 2018 (1)	PIB según Banco Mundial en 2020 (En millones de USD) (2)	PIB per cápita según Banco Mundial en 2020 (En USD) (3)	Población Total en 2021 (4)
**Argentina**	9,6%	383 066,90	8 441,90	44 939 000,00
**Australia**	9,2%	1 330 900,90	51 812,20	25 550 000,00
**Brasil**	9,5%	1 444 733,20	6 796,80	210 147 000,00
**Chile**	9,1%	252 940, 00	13 231,70	19 107 000,00
**Colombia**	7,6%	271 346,90	5 332,80	50 374 000,00
**Cuba**	11, 1%	103 131,00	9 099,70	11 333 483,00
**Estados Unidos**	16,8%	20 936 600,00	63 543,60	328 461 000,00
**Honduras**	7%	23 827,80	2 405,70	9 770 000,00
**Nueva Zelandia**	9,2%	212 482,00	41 791,80	5 090 000,00
**Perú**	5,2%	202 014,30	6 126,90	32 510 453,00
**Uruguay**	9,2%	53 628,80	15 438,4	3 461 734,00
**Venezuela**	3,5%	482 359,30	16 055,60	28 515 829,00

Fuente (1): https://data.worldbank.org/indicator/SH.XPD.CHEX.GD.ZS

Fuente (2): https://datos.bancomundial.org/indicator/NY.GDP.MKTP.CD

Fuente (3): https://datos.bancomundial.org/indicator/NY.GDP.PCAP.CD

Fuente (4): https://datosmacro.expansion.com/demografia/poblacion

**CUADRO 2.** Porcentaje del presupuesto total de Salud Pública, dedicado a Salud Mental en países seleccionados.

País	Porcentaje del gasto en Salud Mental del presupuesto total de Salud	Año reportado en Salud Mental	Fuente
**Argentina**	2%	2017	Primer Congreso Provincial de Salud Mental y Adicciones, Ministerio de Salud de Argentina: https://www.ms.gba.gov.ar/sitios/congresosaludmentalyadicciones/2017/05/13/gasto-en-servicios-de-salud-mental-estudio-en-un-grupo-de-paises-y-comparacion-con-el-caso-argentino/
**Australia**	7,8%	2019	Australian Institute of Health and Welfare: www.aihw.gov.au/reports/mental-health-services/mental-health-services-in-australia/report-contents/expenditure-on-mental-health-related-services
**Brasil**	2,4%	2017	Primer Congreso Provincial de Salud Mental y Adicciones, Ministerio de Salud de Argentina: https://www.ms.gba.gov.ar/sitios/congresosaludmentalyadicciones/2017/05/13/gasto-en-servicios-de-salud-mental-estudio-en-un-grupo-de-paises-y-comparacion-con-el-caso-argentino/
**Chile**	2,4%	2017	Ministerio de Salud de Chile. Plan Nacional de Salud Mental 2017 a 2025.
**Colombia**	El presupuesto no estaría diferenciado del general de salud	2018	Ministerio de Salud (2018). Estructura del gasto en Salud Pública en Colombia.
**Estados Unidos**	5,5%	2020	PR News Wire: https://www.prnewswire.com/news-releases/2019-us-mental-health-spending-topped-225-billion-with-per-capita-spending-ranging-from-37-in-florida-to-375-in-maine--open-minds-releases-new-analysis-301058381.html
**Honduras**	1,8%	2008	Primer Congreso Provincial de Salud Mental y Adicciones, Ministerio de Salud de Argentina: https://www.ms.gba.gov.ar/sitios/congresosaludmentalyadicciones/2017/05/13/gasto-en-servicios-de-salud-mental-estudio-en-un-grupo-de-paises-y-comparacion-con-el-caso-argentino/
**Nueva Zelandia**	6%	2021	Stuff News Paper: https://www.stuff.co.nz/national/politics/300344864/mental-health-just-6-per-cent-of-cash-for-mental-health-facilities-spent
**Perú**	2%	2018	Ministerio de Salud (2018). Plan Nacional de Fortalecimiento de servicios de Salud Mental Comunitaria.
**Uruguay**	7%	2017	Primer Congreso Provincial de Salud Mental y Adicciones, Ministerio de Salud de Argentina: https://www.ms.gba.gov.ar/sitios/congresosaludmentalyadicciones/2017/05/13/gasto-en-servicios-de-salud-mental-estudio-en-un-grupo-de-paises-y-comparacion-con-el-caso-argentino/
**Venezuela**	5%	2013	Organización Mundial de la Salud (2013). Informe sobre el Sistema de Salud Mental en la República Bolivariana de Venezuela utilizando el Instrumento de Evaluación para Sistemas de Salud Mental de la Organización Mundial de la Salud (OMS-IESM).

En cuanto a los contenidos de los distintos programas de salud mental, el 59,7% serían diseñados con base a la evidencia disponible(12).En Latinoamérica, los países han incorporado paulatinamente el enfoque comunitario y los determinantes sociales en sus planes nacionales de salud mental; por ejemplo, Perú (13) y Colombia (14), tienen planes nacionales de salud mental diferenciados del plan general de salud pública. En ambos casos, se mencionan los determinantes sociales como relevantes en la prevalencia de trastornos mentales y la necesidad de fortalecer el sector primario en la atención de salud mental, así como en educar a la comunidad en este ámbito. El programa de Perú por su parte también destaca la necesidad de la intervención temprana en infancia para prevenir el desarrollo de enfermedades mentales y la importancia de descentralizar territorialmente la atención y especialización en salud mental.

En el caso de Chile, el Plan Nacional de Salud Mental 2020-2025 del Ministerio de Salud, incorpora en sus lineamientos la complejidad etiológica de los trastornos mentales, haciendo referencia por ejemplo, a los determinantes sociales de la salud, afirmando que las intervenciones en salud mental deben ser con un enfoque comunitario y de carácter biopsicosocial, enfatizando en la necesidad de intervenir en la calidad y modos de vida de las personas, a fin de favorecer su bienestar, integración social y la promoción de sus derechos humanos (15).

Honduras por su parte, en su plan general de salud pública (16), incorporó un breve apartado de salud mental, indicando determinantes sociales que merman la salud mental de su población, destacando la violencia urbana, homicidios, violencia de género y la situación migratoria. Menciona los derechos humanos de las personas con trastornos mentales y en situación de discapacidad. Presentó datos epidemiológicos de la salud mental en el país, pero sin mayor actualización, los datos corresponden a inicios de la década del 2000. En general, los programas tienden a carecer de estrategias concretas para operacionalizar las necesidades en salud mental de su población.

Los ejemplos anteriores constituyen un avance en cuanto a transitar desde un paradigma biomédico, hacia uno más integral. Sin embargo, todavía existe escasa investigación traslacional en Latinoamérica que ilustre que efectivamente este enfoque basado en la evidencia esté implementándose en los diversos territorios. A diferencia de países de altos ingresos, los países de rentas medias y bajas continúan detentando un porcentaje bajo del presupuesto total de salud para políticas públicas en salud mental, por lo que todavía esta temática no pareciera ser una prioridad para el sistema sanitario, tal como se ilustra en el cuadro 2.

Por otra parte, la sistematización y evaluación de la calidad de la implementación de programas basados en un enfoque comunitario, continúa siendo escasa tanto en países de altos, medianos y bajos ingresos (6).

En cuanto a experiencias de ejecución de políticas públicas basadas en un enfoque comunitario, desde 2014 la reforma holandesa para el abordaje de la salud mental nacional ha propendido a que la atención primaria sea quien aborde los casos de salud mental de baja y mediana gravedad, siendo derivados a la atención terciaria, solo aquellos casos de mayor severidad. Para evaluar la efectividad de ese tránsito desde un modelo de atención especializada hacia uno de atención primaria de la reforma holandesa, Magnée et al. (17) realizaron un estudio en el que aleatoriamente se designaron pacientes a la atención especializada y otros a la práctica general de baja especialización, observándose una reducción estadísticamente significativa en sintomatología asociada a trastornos mentales luego de tres meses, independientemente del tipo de tratamiento asignado, sin exhibir diferencias significativas en los grupos de intervención de salud mental general y aquellos especializados. El estudio concluye que el potencial del tránsito a la atención menos especializada para el abordaje de la salud mental es alto, sin embargo, se requiere de mayor sistematización de los programas, entrenamiento al personal de salud y más investigación dentro de los servicios de salud primaria para un alcanzar abordaje efectivo de la salud mental de la población.

Asimismo, en Nigeria se realizó un estudio en el que se evaluó la transición de un programa local desde el enfoque hospitalario hacia el comunitario mediante el fortalecimiento de la atención en salud mental en la atención primaria, concluyendo que independientemente de la escases de recursos económicos, al capacitar a los profesionales del sistema primario en temáticas básicas de salud mental y realizando operaciones de fortalecimiento y gestión con las redes comunitarias, se podría lograr una transición exitosa hacia este modelo, principalmente en lo relativo del acceso a la atención en salud mental desde el sistema primario(18).

Un ejemplo de operacionalización de las recomendaciones de la OMS en Latinoamérica es Argentina, con el Programa de acción para superar las brechas en salud mental mhGAP en 2019 (19), en el que se adaptaron los lineamientos de la guía original de la Organización Panamericana de la Salud (OPS) al contexto local, mediante mesas de diálogo con profesionales de la salud mental, salud general, representantes de comunidades urbanas y rurales. Se buscó potenciar la salud mental desde un enfoque comunitario, mediante la capacitación a profesionales no especializados en salud mental de los equipos de salud primaria. Ésta instrucción fue desarrollada de manera virtual, buscando incorporar al personal de todo el territorio nacional.

Con relación a los determinantes sociales, muchos de los países de ingresos medios y bajos, todavía enfrentan la problemática de la inequidad y la pobreza. Por ejemplo, Chile se mantiene como uno de los países con mayores niveles de desigualdad dentro de los de La Organización para la Cooperación y Desarrollo Económicos (OCDE) con altas brechas entre los deciles más altos y bajos de la población (18), existiendo todavía un alto índice de pobreza multidimensional (IPM), con una tasa nacional del 20,7% (20).

La pobreza constituye un factor de estrés sistemático que contribuye al desarrollo de trastornos mentales (5), en consecuencia, Chile presenta preocupantes tasas de prevalencia de trastornos de salud mental, constituyendo la depresión y los trastornos por consumo de alcohol aquellos de mayor prevalencia, con tasas del 9,9% y 5,1% respectivamente, tasas que varían también territorialmente a lo largo del país y cuyas variaciones también se asocian a las diversidades socioculturales (21).

Pareciera que, si bien la teoría y los objetivos de los planes nacionales de salud mental de distintos países propenden a una incorporación del enfoque comunitario y biopsicosocial, todavía hay grandes desafíos para la implementación efectiva de este modelo. Se observa un progresivo esfuerzo por parte de diversas naciones en incorporar el enfoque biopsicosocial en el desarrollo de políticas públicas de salud mental y aumentar la evidencia disponible en esta materia. Sin embargo, las realidades socioculturales de los países son distintas y, por lo tanto, sus necesidades también.

Países de altos ingresos disponen de mayores recursos económicos, discusión académica y programas de salud mental que han madurado paulatinamente desde los años 60 (22), pero manteniendo desafíos en la operacionalización de este enfoque en la ejecución de sus programas. También requieren incrementar sus investigaciones para acumular evidencia más conclusiva de la efectividad de sus estrategias de intervención biopsicosocial. Por su parte, las problemáticas de países con bajos y medianos ingresos, guarda relación con la falta de financiamiento; la pobreza en parte importante de su población contribuye al desarrollo de psicopatología, dado que ésta merma la calidad de vida de las personas y las expone a factores de riesgo que aumentan su probabilidad de enfermedad mental, incluso desde la infancia temprana (23). Particularmente, América Latina exhibe brechas en el acceso al sistema sanitario, tanto en la cantidad como en la calidad de sus servicios, presentando estos países, también grandes diferencias entre ellos mismos, tanto en sus prevalencias psiquiátricas, como en investigación y en sus políticas públicas (24).

En conclusión, la evidencia sugiere el abordaje biopsicosocial de las problemáticas de salud mental, mediante el fortalecimiento del sistema primario e intervenciones de carácter comunitario, así como la incorporación de los determinantes sociales en la estrategia programática. Pareciera que, en países de ingresos altos los desafíos guardarían relación principalmente con transitar desde la práctica hospitalocéntrica hacia la intervención primaria y comunitaria, debido a los desafíos en la implementación de las nuevas políticas, requiriéndose incrementar la evidencia respecto a la eficacia de los programas basados en este enfoque.

En los países de ingresos medios y bajos, las necesidades serían todavía más básicas y guardarían relación primordialmente con las brechas en el acceso y el bajo financiamiento para la salud mental, así como de la todavía somera experiencia en la implementación de políticas públicas integrales e intersectoriales. Por tanto, la evidencia indica que para virar desde un enfoque biomédico hacia uno que incorpore los determinantes sociales de la salud, no se deberían mantener idénticas estrategias en comunidades distintas, puesto que probablemente no serían efectivas para responder a sus necesidades particulares.

Los resultados exponen que hay un avance en países de ingresos medios y bajos, particularmente de Latinoamérica, para desarrollar programas de salud mental e incorporar en éstos una perspectiva comunitaria y biopsicosocial. Sin embargo, se presentan desafíos en su operacionalización, financiamiento y adaptación a sus realidades culturales y territoriales. De esta manera, si bien existe un aumento en la mención a la necesidad de abordar la salud mental y sus determinantes sociales, hay países como Honduras, que lo aluden sucintamente en un apartado del programa general de salud y otros como Perú, Colombia y Chile, que presentan un plan nacional de salud mental diferenciado al de salud general.

Respecto a cómo ejecutar los objetivos de la política pública, los programas no presentan mayor detalle de estrategias concretas y en algunos casos, no especifican qué porcentaje de recursos del presupuesto total de salud será destinado a salud mental. En aquellos casos que sí lo exponen, este porcentaje tiende a mantenerse muy bajo, entre el 2% y 3%, en comparación con países de ingresos altos, que tienden a mantener un presupuesto por sobre el 6% del total del gasto en salud.

En lo relativo a la investigación en salud mental, durante las últimas dos décadas ésta aumentó en países de ingresos bajos, sin embargo, la incorporación del enfoque biopsicosocial en los estudios ha sido paulatina, tendiendo a mantener una escasa productividad y actualización de datos respecto del análisis de la efectividad y operacionalización de estas políticas públicas. Aun así, hay estudios que dan cuenta de experiencias de implementación de modelos de potenciación de la salud primaria, como es el caso de Argentina y Nigeria, pero la evidencia proviene principalmente en países de renta alta. No obstante, países de ingresos bajos y medios, tienden a concluir sobre la necesidad de descentralizar el acceso y la especialización en salud mental concentrada principalmente en las grandes urbes y proponen gestionar eficientemente el capital humano y comunitario para responder a las necesidades territoriales, más allá de los recursos económicos disponibles.

Por lo tanto, existe un creciente reconocimiento del impacto de los determinantes sociales en la salud mental y de la necesidad de abordar los desafíos asociados desde políticas públicas integrales que incorporen el modelo biopsicosocial, lo cual se ve reflejado progresivamente en algunos países latinoamericanos. Nos encontramos en un periodo de transición, en el que se enfrentan los desafíos prácticos y técnicos de efectivamente implementar este paradigma-teórico, con toda la complejidad que aquello implica en la práctica.

Finalmente, recomendamos que, dada la alta prevalencia de trastornos psiquiátricos a nivel mundial, la salud mental debiese ser entendida como un problema social y de salud pública en las distintas naciones, consideraciones que progresivamente han incorporado países latinoamericanos en la planificación de sus políticas públicas. Sin embargo, naciones de ingresos medios y bajos mantienen el gran desafío de avanzar desde los lineamientos programáticos, hacia medidas concretas de financiamiento y operacionalización de los objetivos planteados según su realidad sociocultural.

Para hacerse cargo de los determinantes sociales de la salud, principalmente en países con niveles significativos de inequidad y pobreza, una política pública que realmente comprenda el impacto de los modos de vida y la calidad de vida en el estado psicoemocional de las personas necesariamente debiese coordinar distintos estamentos de la sociedad y no intervenir aisladamente. En países de bajos recursos económicos, la gestión eficiente de distintos actores e instituciones podría favorecer el desarrollo de políticas públicas más integrales y efectivas, puesto que aspectos como la educación, la cultura, el deporte, el medioambiente y la recreación, influyen significativamente en los determinantes sociales y niveles de bienestar de las personas, estrategias que no se ve claramente reflejada en los programas de salud mental en países latinoamericanos. En esa línea, políticas públicas que no aborden sistémicamente las inequidades, la pobreza, serán siempre insuficientes para enfrentar la epidemiología de los trastornos mentales.

Asimismo, resulta fundamental que los países aumenten la investigación en salud mental y en sistematizar las evaluaciones de sus programas, dado que la réplica de intervenciones utilizadas en países de altos ingresos no necesariamente tendrá un impacto similar en países en vías de desarrollo. Para clarificar las necesidades reales de las distintas comunidades nacionales y locales, investigaciones de carácter local pueden aportar información relevante para diseñar programas de salud mental que efectivamente respondan a las necesidades de su población.

## Declaración.

Las opiniones expresadas en este manuscrito son responsabilidad de los autores y no reflejan necesariamente los criterios ni la política de la *RPSP/PAJPH* o de la OPS

## References

[1] 1. Lemmi, V. Sustainable development for global mental health: a typology and systematic evidence mapping of external actors in low-income and middle-income countries. BMJ Glob Health. 2019; 4(6): e001826. 10.1136/bmjgh-2019-001826 PMC6936513 31908860

[2] 2. World Health Organization. Mental health [Internet]. www.who.int. 2019. [Citado el 10 de mayo 2021] Disponible en: https://www.who.int/health-topics/mental-health#tab=tab_1

[3] 3. 1.World Health Organization. Social determinants of health [Internet]. World Health Organisation. WHO; 2020. [Citado el 15 de mayo 2021] Disponible en: https://www.who.int/health-topics/social-determinants-of-health#tab=tab_1

[4] 4. Knifton L., Inglis G. Poverty and mental health: policy, practice and research implications. BJPsych Bull. 2020; 44(5): 193-196. 10.1192/bjb.2020.78 PMC7525587 32744210

[5] 5. Costello EJ, Compton SN, Keeler G, Angold A. Relationships Between Poverty and Psychopathology. JAMA. 2003; 290(15): 2023. 10.1001/jama.290.15.2023 14559956

[6] 6. Saraceno B. Discurso global, sufrimientos locales. Análisis crítico del Movimiento por la Salud Mental Global. Herder, Barcelona; 2018.

[7] 7. Atkinson JA, Skinner A, Lawson K, Rosenberg S, Hickie IB. Bringing new tools, a regional focus, resource-sensitivity, local engagement and necessary discipline to mental health policy and planning. BMC Public Health. 2020; 20(1): 1-10. 10.1186/s12889-020-08948-3 PMC7273655 32498676

[8] 8. World Health Organization. Mental health action plan 2013 - 2020 2013-2020. [Internet] Geneva: WHO; 2021 [Citado el 20 mayo 2021] Disponible en: https://www.who.int/publications/i/item/9789241506021

[9] 9. Zhou W, Yu Y, Yang M, Chen L, Xiao S. Policy development and challenges of global mental health: a systematic review of published studies of national-level mental health policies. BMC Psychiatry. 2018; 18(1): 5-15. 10.1186/s12888-018-1711-1 PMC5960139 29776356

[10] 10. Wainberg ML, Scorza P, Shultz JM, Helpman L, Mootz JJ, Johnson KA, Arbuckle MR. Challenges and Opportunities in Global Mental Health: a Research-to-Practice Perspective. Curr Psychiatry Rep. 2017; 19(5): 5-15. 10.1007/s11920-017-0780-z PMC5553319 28425023

[11] 11. Misra S, Stevenson A, Haroz EE, de Menil V, Koenen KC. ‘Global mental health’: systematic review of the term and its implicit priorities. BJPsych Open. 2019; 5(3): 1-8. 10.1192/bjo.2019.39 PMC6582218 31530316

[12] 12. Hui A, Rains LS, Todd A, Boaz A, Johnson S. Correction to: The accuracy and accessibility of cited evidence: a study examining mental health policy documents. Soc Psychiatry and Psychiatr Epidemiol. 2019; 55(1): 123. 10.1007/s00127-019-01802-x 31709459

[13] 13. Ministerio de Salud. Plan Nacional de Fortalecimiento de servicios de Salud Mental Comunitaria. Lima: Ministerio de Salud; 2018.

[14] 14. Ministerio de Salud y Protección Social. Política Nacional de Salud Mental. Bogotá: Ministerio de Salud y Protección Social; 2018

[15] 15. Ministerio de Salud de Chile. Plan Nacional de Salud Mental 2017 a 2025. [Internet] Santiago de Chile: Ministerio de Salud de Chile;2021 [Citado el 30 junio 2021). Disponible en: https://www.minsal.cl/wp-content/uploads/2017/12/PDF-PLAN-NACIONAL-SALUD-MENTAL-2017-A-2025.-7-dic-2017.pdf

[16] 16. Secretaría de Estado en el Despacho Salud & Instituto Hondureño de Seguridad Social. Plan Nacional de Salud 2021.Tegucigalpa: Secretaría de Estado en el Despacho Salud & Instituto Hondureño de Seguridad Social; 2020.

[17] 17. Magnée T, de Beurs DP, Kok TY, Verhaak PF. Exploring the feasibility of new Dutch mental health policy within a large primary health care centre: a case study. Family Practice. 2017; 35(2): 186-192. 10.1093/fampra/cmx084 28973383

[18] 18. Ryan GK, Nwefoh E, Aguocha C, Ode PO, Okpoju SO, Ocheche P, Woyengikuro A, ABdilmalik J, Eaton J. Partnership for the implementation of mental health policy in Nigeria: a case study of the Comprehensive Community Mental Health Programme in Benue State. Int J Ment Health Syst. 2020; 14(1): 1-13. 10.1186/s13033-020-00344-z PMC7033947 32110245

[19] 19. Dirección Nacional de Salud y Adicciones. Implementación del Programa MHGAP en Argentina. Fundación Superación de la Pobreza. [Internet] [Citado 7 julio de 2021]. Buenos Aires: Dirección Nacional de Salud y Adicciones; 2019 Disponible en http://www.superacionpobreza.cl/quienes-somos/

[20] 20. Ministerio de Desarrollo Social Chile. Encuesta Casen. [Internet]Santiago de Chile: Ministerio de Desarrollo Social Chile. Encuesta Case; 2021 [Citado 02 julio de 2021] Disponible en: http://observatorio.ministeriodesarrollosocial.gob.cl/casen-multidimensional/casen/docs/Resultados_pobreza_Casen_2017.pdf

[21] 21. Vicente B, Kohn R, Saldivia S, Rioseco P. Carga del enfermar psíquico, barreras y brechas en la atención de salud mental en Chile. Rev Med Chil. 2007; 135: 1630-1638. 18357362

[22] 22. Benjet C. La salud mental de la niñez y la adolescencia en América Latina y el Caribe. Epidemiología de los trastornos mentales en América Latina y el Caribe. 2009; 234-242.

[23] 23. Benjet C. La salud mental de la niñez y la adolescencia en América Latina y el Caribe. Epidemiología de los trastornos mentales en América Latina y el Caribe. 2009; 234-242.

[24] 24. Kohn R, Rodriguez J. Prevalencia y carga de los trastornos mentales en la población adulta de América Latina y el Caribe. Epidemiología de los trastornos mentales en América Latina y el Caribe. 2009; 19-34.

